# Cytokine Profile of Children Hospitalized with Virologically-Confirmed Dengue during Two Phase III Vaccine Efficacy Trials

**DOI:** 10.1371/journal.pntd.0004830

**Published:** 2016-07-26

**Authors:** Anke Harenberg, Aymeric de Montfort, Frédérique Jantet-Blaudez, Matthew Bonaparte, Florence Boudet, Melanie Saville, Nicholas Jackson, Bruno Guy

**Affiliations:** 1 R&NCS Department, Sanofi Pasteur, Marcy l’Etoile, France; 2 Global Clinical Immunology Department, Sanofi Pasteur, Swiftwater, Pennsylvania, United States of America; 3 Global Clinical Development, Sanofi Pasteur, Marcy l’Etoile, Lyon, France; 4 Research and Development, Sanofi Pasteur, Lyon, France; Oxford University Clinical Research Unit, VIETNAM

## Abstract

**Background:**

Two large-scale efficacy studies with the recombinant yellow fever-17D–dengue virus, live-attenuated, tetravalent dengue vaccine (CYD-TDV) candidate undertaken in Asia (NCT01373281) and Latin America (NCT01374516) demonstrated significant protection against dengue disease during two years’ active surveillance (active phase). Long-term follow up of participants for breakthrough disease leading to hospitalization is currently ongoing (hospital phase).

**Methodology/Principal findings:**

We assessed the cytokine profile in acute sera from selected participants hospitalized (including during the active phase) up to the beginning of the second year of long-term follow up for both studies. The serum concentrations of 38 cytokines were measured in duplicate using the Milliplex Human Cytokine MAGNETIC BEAD Premixed 38 Plex commercial kit (Millipore, Billerica, MA, USA). Partial least squares discriminant analyses did not reveal any difference in the overall cytokine profile of CYD-TDV and placebo recipients hospitalized for breakthrough dengue regardless of stratification used. In addition, there was no difference in the cytokine profile for breakthrough dengue among those aged <9 years versus those aged ≥ 9 years.

**Conclusions/Significance:**

These exploratory findings show that CYD-TDV does not induce a particular immune profile versus placebo, corroborating the clinical profile observed.

## Author Summary

A live-attenuated, tetravalent dengue vaccine (CYD-TDV) has been shown to provide protection against dengue disease in two large-scale, placebo-controlled, phase III efficacy studies. Continued surveillance of study participants was subsequently undertaken to better define longer term vaccine efficacy and safety. A yet unexplained higher incidence of hospitalization for dengue disease was observed among children aged <9 years in year 3 of follow up. While the clinical outcome of the hospitalized cases was similar between CYD-TDV and placebo recipients, it was important to further investigate whether the immune profile induced by breakthrough infection differed between the two study groups. We compared the profile of 38 cytokines, chemokines and growth factors in acute phase sera collected from participants with breakthrough disease in the two groups. No difference in overall profile was observed between CYD-TDV and placebo recipients. Similarly, no difference in the cytokine profile for breakthrough dengue was observed between those aged <9 years and those aged ≥ 9 years. Based on these analyzed factors, our study shows that CYD-TDV does not induce an overall altered immunological profile with breakthrough disease compared with placebo, in agreement with the similar clinical pictures and viremia observed in the two groups.

## Introduction

Dengue virus (DENV) is the most important mosquito-borne pathogen threatening approximately half of the world’s population, mostly in tropical and subtropical areas including Latin America and Southeast Asia [1,2]. Infection with any of the four DENV serotypes can be asymptomatic or cause a spectrum of clinical symptoms from mild fever (dengue fever/DF) to more severe, potentially life-threatening disease including dengue hemorrhagic fever and shock syndrome (DHF/DSS) [3]. Severe dengue is most often observed in previously infected subjects undergoing secondary infection with a different DENV serotype [4]. DHF/DSS is associated with excessive immune activation, or a ‘cytokine storm’, which may contribute to increased vascular permeability with extensive plasma leakage and resultant signs of shock [5,6]. Differences in the cytokine profile in severe versus non-severe disease have been demonstrated [7–10].

Innate and adaptive immune responses have been proposed to contribute to the cytokine profile and disease outcome [11]. Firstly, in the humoral response to DENV infection, antibody-dependent enhancement of infection (ADE) by preexisting “enhancing” antibodies may play a role in the development of severe disease with second or subsequent infection with heterotypic dengue serotypes [12]. “Intrinsic ADE” could also occur through production of the immunosuppressive IL-10 and proinflammatory mediators, including IL-6 and TNF-α, responsible in part for increased vascular permeability [13,14]. Secondly, in the cell-mediated immune response, several studies suggest involvement of T-cells in the ‘original antigenic sin’ phenomenon in severe dengue. According to this hypothesis, inappropriate low avidity cross-reactive T-cells induced by a secondary heterotypic infection may produce an altered cytokine profile, including increased production of inflammatory cytokines, such as TNF-α and CCL4/MIP1β, and decreased production of IFN-γ and IL-2 [11].

There are currently no vaccines or antiviral therapies available for the management of dengue disease. Several vaccine formulations are in development [15], and one has recently obtained marketing authorization in several countries, Dengvaxia, the recombinant yellow fever-17D–dengue virus, live-attenuated, tetravalent dengue vaccine (CYD-TDV). Two large-scale phase III efficacy studies have recently been conducted with CYD-TDV in Asia and Latin America, demonstrating significant protection against dengue disease [16,17]. Continued surveillance of participants from these two studies for breakthrough disease leading to hospitalization is ongoing to better define long-term vaccine efficacy and safety. After 3 years, the cumulative risk of hospitalization among children aged 2–16 years was still lower in the CYD-TDV group than the control group; however, an unexplained higher incidence of hospitalization for dengue disease was observed among children aged <9 years in year 3 [18]. While the clinical outcome of these hospitalized/severe cases was similar between CYD-TDV and placebo recipients, with all cases fully recovered following supportive medical care, it was important to investigate further if the immune profile induced after infection could differ between the two treatment groups. We assessed 38 cytokines in sera collected during the acute phase of breakthrough disease among CYD-TDV and placebo recipients from the two phase III trials.

## Methods

### Participants and samples

Details of the clinical protocols (ClinicalTrials.gov numbers: NCT01374516 and NCT01373281) and study population are described elsewhere [16–18]. Briefly, eligible participants aged 2–14 years in the Latin American study (CYD15) and 9–16 years in the Asian study (CYD14) were randomized (2:1) to receive CYD-TDV or placebo (0.9% NaCl). After the initial active surveillance phase (active phase) for breakthrough dengue (up to 25 months after the first injection), a four-year long-term follow-up phase was initiated to monitor breakthrough disease leading to hospitalization (hospital phase). Participants attended yearly clinic visits, with regular contact with study personnel between visits (at least every 3 months). Hospitalization for acute fever was recorded during study contacts and visits, and by self-reporting and surveillance of non-study hospitals. As a post-hoc exploratory analysis, we tested blood samples from selected participants at one month post-dose 3 for baseline cytokine assessment, and during the acute phase of febrile illness from participants who required hospitalization for virological confirmation of dengue infection in the active phase, and during the first and beginning of the second year of long-term follow up. The cut-off for the current analysis was 28 November 2014. During the acute phase, apart from a few cases, samples were obtained between day 1 (D1) and D7 after fever onset, and no difference was observed in the daily distribution of samples between the CYD-TDV and placebo groups in this interval of time (p = 0.99, Kuiper test).

### Ethics statement

The trials were undertaken in compliance with good clinical practice guidelines and the principles of the Declaration of Helsinki. Ethics review committees approved the protocol and subsequent amendments as well as the consent and assent forms. Parents or legal guardians provided written informed consent for all children, and older children also signed informed-assent forms, in compliance with the regulations of each country. For the exploratory study reported herein, the laboratory was blinded to treatment group during sample assessments and unblinded in order to perform the statistical analysis.

### Quantification of cytokines

We evaluated 38 cytokines: sCD40L (sCD154), EGF, eotaxin/CCL11, FGF-2/FGF-basic, Flt3 Ligand, Fractalkine/CX3CL1, G-CSF, GM-CSF, GRO (isoforms GROα (CXCL1), GROβ (CXCL2), and GROγ (CXCL3)), IFN-α2, IFN-γ, IL-1α, IL-1β, IL-1Ra, IL-2, IL-3, IL-4, IL-5, IL-6, IL-7, IL-8/CXCL8, IL-9, IL-10, IL-12 (p40), IL-12 (p70), IL-13, IL-15, IL-17A/CTLA8, IP-10/CXCL10, MCP-1/CCL2, MCP-3/CCL7, MDC/CCL22, MIP-1α/CCL3, MIP-1β/CCL4, TGFα, TNF-α, TNF-β, and VEGF-A. Serum samples were tested undiluted and in duplicate (baseline samples were tested once). Serum cytokine levels were analyzed using the Milliplex Human Cytokine MAGNETIC BEAD Premixed 38 Plex commercial kit according to the manufacturer’s instructions. The fluorescent signals were detected using the multiplex array reader Bio-Plex 200 System. Raw data were initially measured as relative fluorescence intensity and then converted to cytokine concentrations in pg/mL based on the standard curve generated from the reference concentrations supplied with the kit. Means of the duplicates were reported.

Because the levels of two factors—CXCL10/IP-10 and sCD40L —were above the upper limit of the test in a significant number of samples, these factors were measured again separately using dedicated kits for 199 out of 207 samples (for the remaining 8 samples there was not enough volume left to re-run them: 4 in the CYD-TDV (sampled at day 3 (D3), D4, D5 and D7) and 4 in the placebo group (all sampled at D1). Samples were used at 1/10 dilution, except when those values were still above the upper limit, in which case 1/100 dilution was used. The Milliplex Human Cytokine MAGNETIC BEAD 2 Plex custom kit was used according to the manufacturer’s instructions. Cytokine concentrations were measured by the same analytical method as that described above.

### Statistical analysis

For each cytokine, the mean concentration in pg/mL was log_10_ transformed. All comparisons were performed using a Student test if the normality hypothesis of both groups was accepted (Shapiro Wilks test or Kolmogorov test, depending on the sample size). If the normality hypothesis of one or both groups was not accepted, the Wilcoxon non-parametric test was used. In accordance with data distribution, geometric means were considered when comparisons were performed using a Student test, and medians were considered with the Wilcoxon non-parametric test.

The comparisons between the CYD-TDV and placebo groups were stratified as follows: i) all hospitalized cases, irrespective of phase or severity; ii) severe or non-severe cases only; iii) active phase or hospital phase only; iv) cases aged <9 years or aged ≥9 years. Additionally, the profile of overall cases (CYD-TDV and placebo groups combined) was compared between severe and non-severe cases. For cytokines with a significant difference between the CYD-TDV and placebo groups, an analysis of variance was conducted to evaluate whether the difference was study- or group-dependent.

The distribution of samples obtained between day 1 and day 7 after fever onset were compared between CYD-TDV and placebo groups using a Kuiper test.

Partial least squares discriminant analyses (PLS-DA) were performed for cytokines that appeared to be differentially expressed between baseline and acute phase of dengue illness to analyze the overall immune profile between: i) the CYD-TDV and placebo groups, ii) CYD14 and CYD15 studies, and iii) the two age groups (<9 years or ≥9 years). The goal was to provide a dimension reduction to relate a binary response variable (the 3 situations described above) to a set of predictor variables (the cytokines). The PLS-DA results give percentage discrimination between the two tested groups, ranging from 0% (no discrimination) to 100% (perfect discrimination).

All comparisons were performed using SAS v9.2 software. The PLS-DA was done with SIMCA-P 9. Margins of error were 5% for effects of the main factors and 1% for normality tests.

## Results

Of the 265 breakthrough cases occurring up to 28 November 2014, 207 serum samples (with sufficient volume for analysis) were available for immune profile assessment (S1 Table). Of these, 24 and 74 in the CYD-TDV group were from cases classified as severe and non-severe, respectively, as defined by the independent data monitoring committee, and 28 and 80, respectively, in the placebo group. Information regarding severity of illness was missing for one sample. An additional 40 samples were taken one month post-dose 3 (17 and 23 in the CYD-TDV and placebo groups, respectively) to assess baseline cytokine levels.

### Immune profile in hospitalized participants compared to baseline

**Fig 1:**
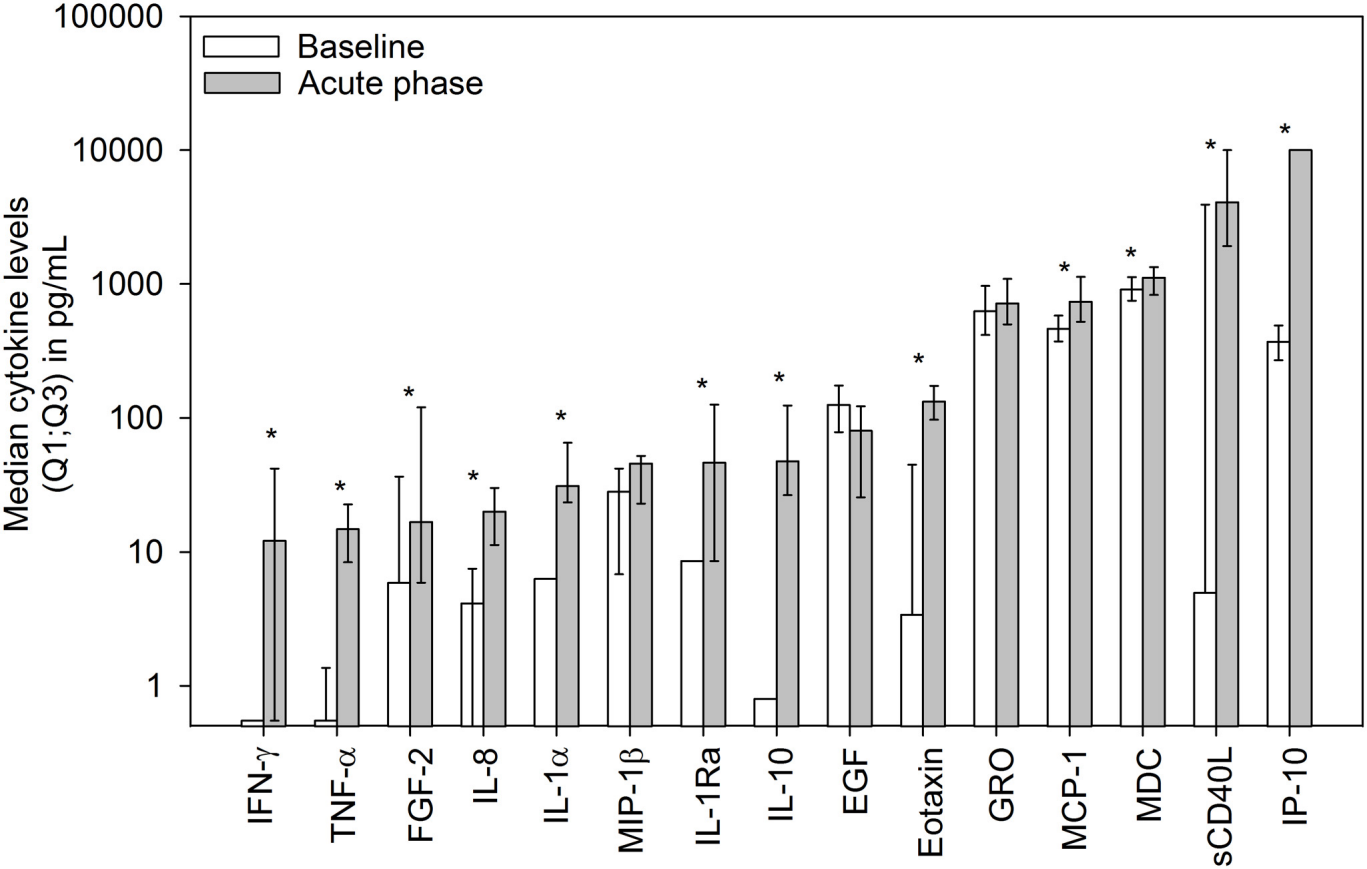
Differentially expressed cytokines in acute samples as compared to baseline. Median cytokine values in children at baseline (1 month post-dose 3) (N = 40) and during the acute phase of dengue illness (N = 33) irrespective of treatment group. Upper error bars represent the 75% (Q3) percentile; the lower error bars represent the 25% (Q1) percentile. Cytokines with a significant difference (p<0.05) are marked with an asterisk. Values below the lower limit of quantification were replaced by half of the limit of quantification.

Of the 40 baseline samples, 33 could be paired with acute-phase samples. At baseline, only 7 cytokines had median levels above the lower limit of quantification (LLOQ): IP-10, MCP-1, MDC, MIP-1β, EGF, GRO and IL-8. In the acute phase, an additional 8 cytokines had median levels above the LLOQ: IFN-γ, TNF-α, FGF-2, IL-1α, IL-1Ra, IL-10, eotaxin, and sCD40L. A paired comparison of these 15 cytokines (irrespective of the treatment group) showed that 12 (eotaxin, FGF-2, IFN-γ, IL-10, IL-1Ra, IL-1α, IL-8, IP-10, MCP-1, MDC, TNF-α and sCD40L) were differentially expressed between baseline and acute phase of dengue illness (Fig 1).

### Overall immune profile

A two dimension PLS-DA of all acute samples (n = 207) was performed on 12 cytokines that were differentially expressed between baseline and the acute phase. The 12 cytokines were plotted to compare samples between the CYD-TDV and placebo groups (S1A Fig), and between the CYD14 and CYD15 studies (S1B Fig). The percentage discrimination between CYD-TDV and placebo groups was 4.5% and between the CYD14 and CYD15 studies, 23.6%. These results suggest that there is no difference in the global cytokine profile between the two study groups or between the two studies. The data from the two studies were thus pooled hereafter.

### CYD-TDV versus placebo for all hospitalized cases

**Fig 2:**
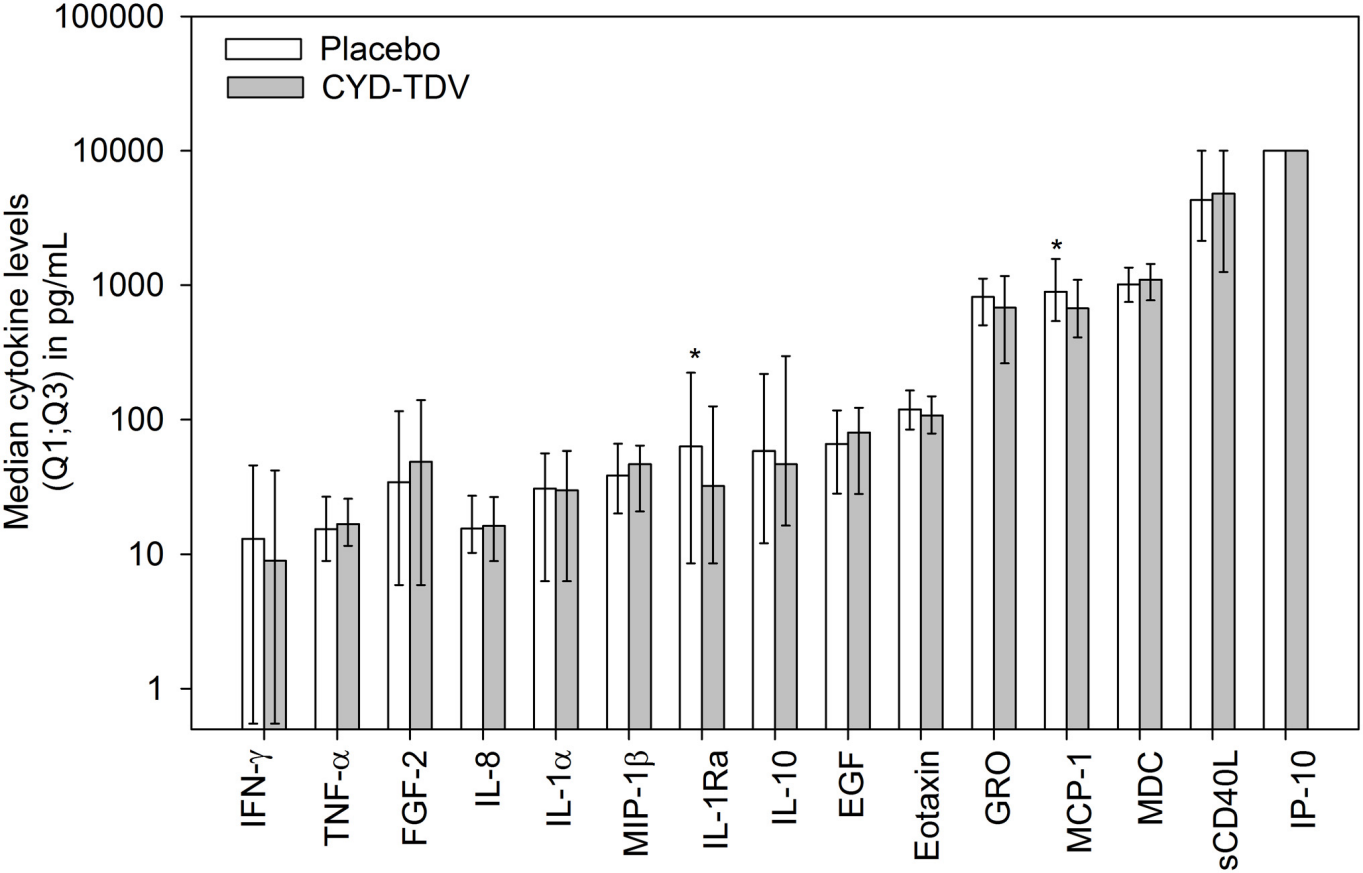
IL-1Ra and MCP-1 levels are significantly different between groups. Median cytokine values in children during the acute phase of dengue illness in CYD-TDV (N = 99) and placebo (N = 108) recipients. Upper error bars represent the 75% (Q3) percentile; the lower error bars represent the 25% (Q1) percentile. Cytokines with a significant difference (p<0.05) are marked with an asterisk. Values below the limit of quantification were replaced by half of the limit of quantification.

Median levels for the 15 cytokines (described earlier) in the CYD-TDV (n = 108) and placebo (n = 99) groups are summarized in Fig 2 for all hospitalized cases. Differences in IP-10 levels in acute samples could not be analyzed since the majority of samples from both CYD-TDV and placebo recipients were levels above the upper limit of the assay (>10,000 pg/mL). Therefore, samples were re-run for this chemokine in a second experiment; as one could not directly compare data obtained in these new analyses to the ones obtained in the first experiment, levels and comparisons based on the new quantifications are presented in S2 Table. In summary, no difference was observed between the two groups for hospitalized cases.

**Fig 3:**
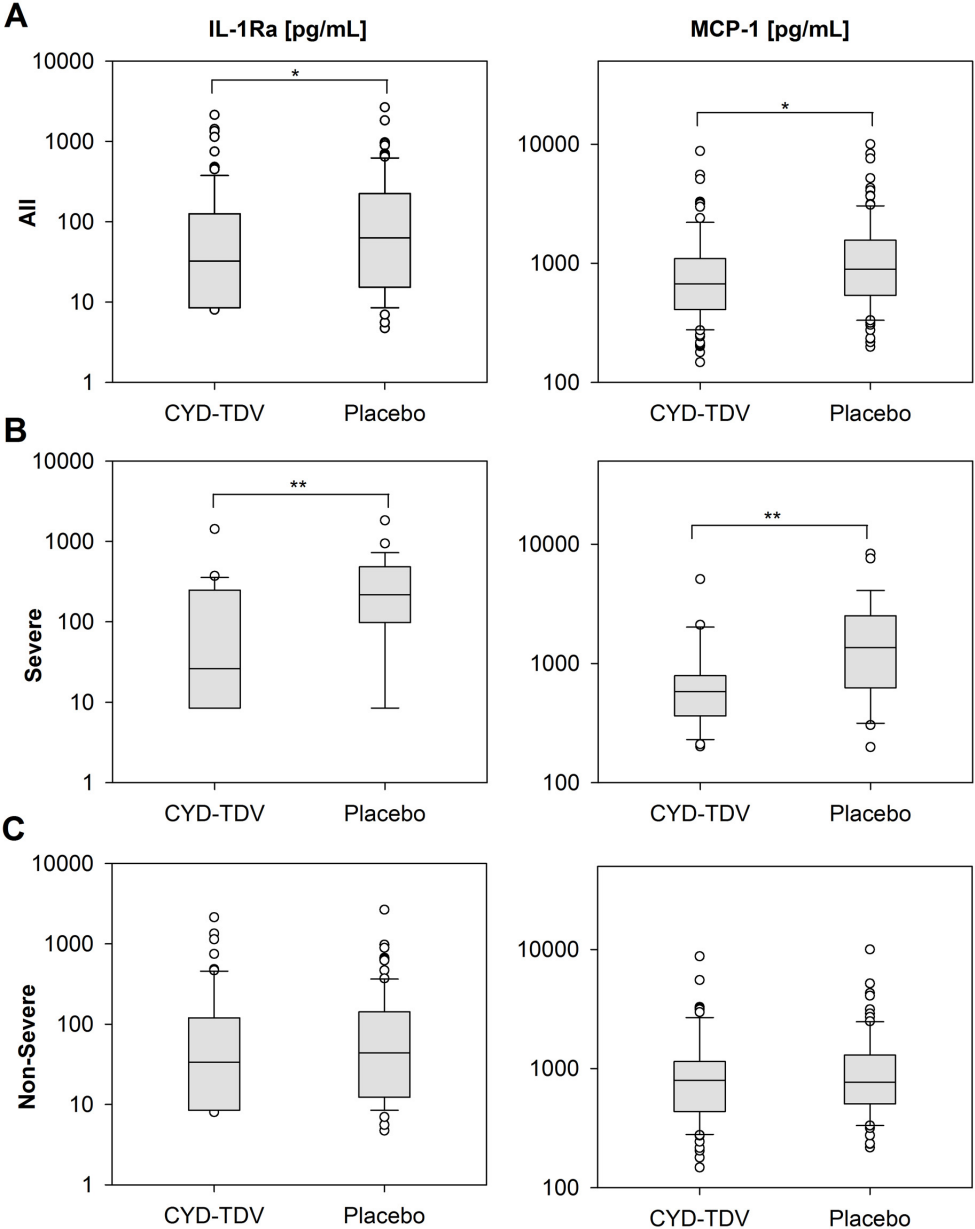
IL-1Ra and MCP-1 levels are significantly different between groups in severe cases. Box and whisker plots of circulating IL-1Ra (left panel) and MCP-1 (right panel) levels during the acute phase of dengue illness in CYD-TDV and placebo recipients: (A) irrespective of severity (CYD-TDV N = 99, Placebo N = 108); (B) in severe cases (CYD-TDV N = 24, Placebo N = 28); and (C) in non-severe cases (CYD-TDV N = 74, Placebo N = 80). The boundary of the box closest to zero indicates the 25th percentile, the line within the box marks the median, and the boundary of the box farthest from zero indicates the 75th percentile. Whiskers (error bars) above and below the box indicate the 90th and 10th percentiles. The individual points represent outliers. Values below the lower limit of quantification were replaced by half of the limit of quantification. Significant differences are indicated by *p < 0.05 and **p ≤ 0.01.

Of the remaining cytokines, only two (IL-1Ra and MCP-1) were significantly different between CYD-TDV and placebo groups—both were higher in the placebo group (median levels for IL-1Ra, 32.30 pg/mL vs. 63.11 pg/mL, respectively [p = 0.038]; geometric means for MCP-1, 735.47 pg/mL vs. 957.94 pg/mL [p = 0.021]; Fig 3A). No differences were observed for these two cytokines between the two groups at baseline (p-values of 0.984 and 0.366 for MCP-1 and IL-1Ra respectively). There was no significant effect of study on IL-1Ra (p = 0.419) or MCP-1 levels (p = 0.064). Thus the difference between the CYD-TDV and placebo groups for these two cytokines was observed equally in both studies. There were no differences in IL-1Ra or MCP-1 levels between the two study groups with respect to the day after fever onset. Both cytokines peaked on the first day after fever onset and declined to baseline levels in the subsequent days (S2A and S2B Fig). The kinetics of sCD40L showed that the levels were similar between day 1 and day 3, declined slightly but stayed at elevated levels until day 7 (S2C Fig).

### Severe versus non-severe cases by treatment group

For the severe cases, IFN-α2, IL-15, IL-1Ra, IP-10 and MCP-1 levels were all significantly lower in the CYD-TDV group (n = 24) than in the placebo group (n = 28). Most participants had IP-10 levels above the upper limit of quantification of the assay preventing a meaningful comparison of data between the two study groups. As stated above, a new analysis was done for this factor, resulting in the same conclusion: significantly lower levels of IP-10 were observed in CYD-TDV than in the placebo group for severe cases (median levels 21648 pg/mL vs 40288 pg/mL, p = 0.033; see S2 Table). Median levels of IL-1Ra for severe cases were 25.4 pg/mL vs. 216.4 pg/mL for CYD-TDV and placebo groups, respectively (p = 0.008), and geometric means of MCP-1 were 600.8 pg/mL vs. 1325.2 pg/mL (p = 0.002) (Fig 3B). For IL-15, these values were 0.9 pg/mL vs. 5.8 pg/mL (p = 0.004) in the two groups, respectively, and for IFN-α2 were 2.4 pg/mL vs. 20.9 pg/mL (p = 0.021). However, IL-15 and IFN-α2 were not significantly different when all cases irrespective of severity were compared; nevertheless, the levels of these cytokines were generally low or undetectable, regardless of group (S3 Fig).

There was no difference in IL-1Ra and MCP-1 levels between non-severe cases in the two study groups (Fig 3C): the median levels of IL-1Ra were 33.5 pg/mL vs. 43.9 pg/mL (p = 0.530) for CYD-TDV and placebo groups, respectively, and the geometric mean levels of MCP-1 were 786.7 pg/mL vs. 855.1 pg/mL (p = 0.513). Since no differences in cytokine profile between the CYD-TDV and placebo groups were found in non-severe cases, the differences found previously for all hospitalized cases (Fig 3A) were due to higher IL-1Ra and MCP-1 levels in the placebo group for severe cases.

### Severe versus non-severe cases irrespective of treatment group

**Fig 4:**
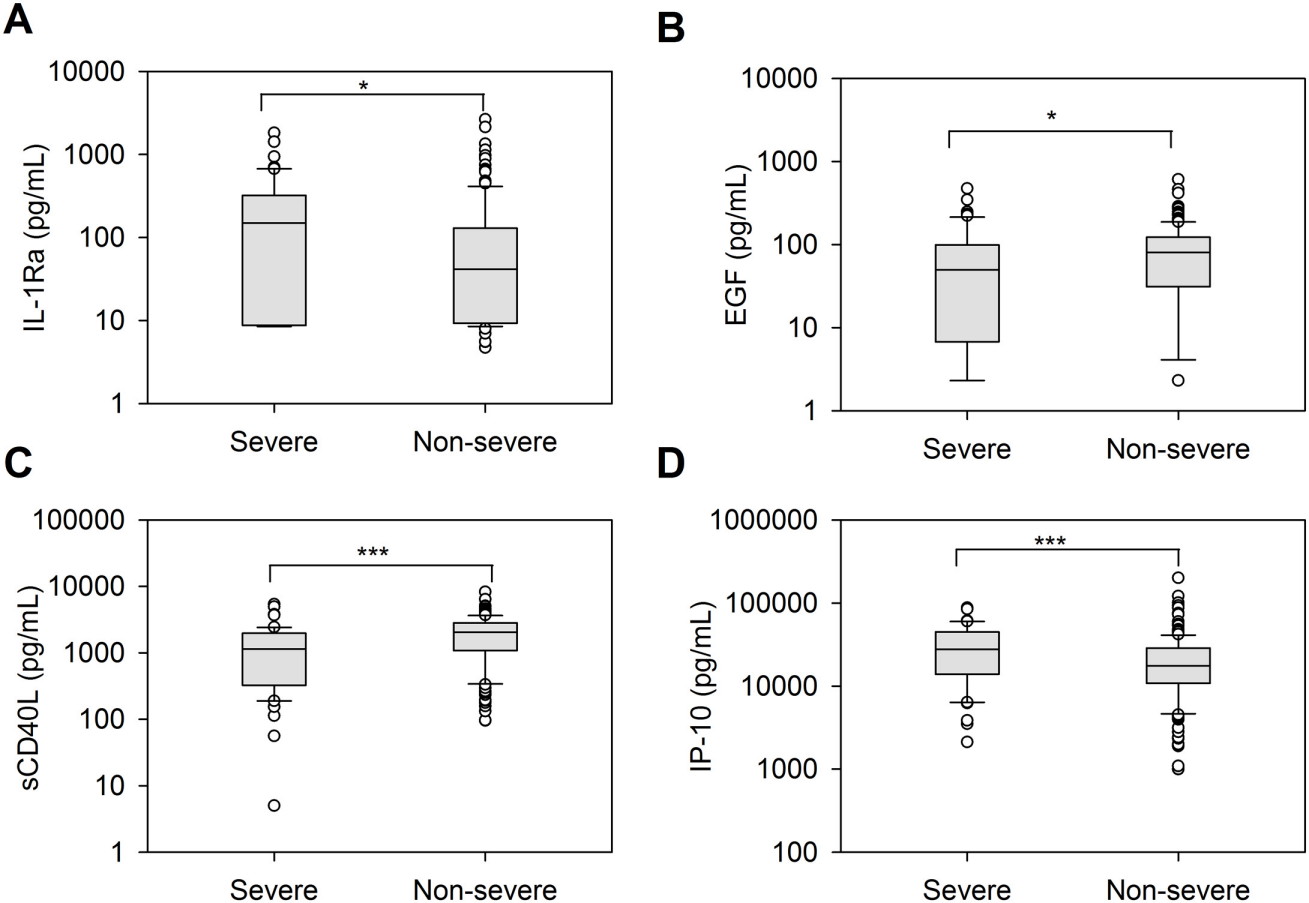
Higher IL-1Ra and IP-10 and lower EGF and sCD40L levels in severe cases. Box and whisker plots of circulating IL-1Ra (A), EGF (B), sCD40L (C) and IP-10 (D) levels during the acute phase of dengue illness in severe (N = 52 for IL-IRA and EGF, N = 50 for sCD40L and IP-10) and non-severe (N = 154 for IL-IRA and EGF, N = 148 for sCD40L and IP-10) cases irrespective of the treatment group. The boundary of the box closest to zero indicates the 25th percentile, the line within the box marks the median, and the boundary of the box farthest from zero indicates the 75th percentile. Whiskers (error bars) above and below the box indicate the 90th and 10th percentiles. The individual points represent outliers. Values below the lower limit of quantification were replaced by half of the limit of quantification. Significant differences are indicated by *p < 0.05 and ***p ≤ 0.001.

PLS-DA could not discriminate between the overall immune profile of severe and non-severe cases (discrimination index, 10.3%). However, when compared individually, EGF, IL-1Ra, and sCD40L were significantly different between severe and non-severe cases, with IL-1Ra the only cytokine higher in severe than in non-severe cases (median levels, 147.8 pg/mL vs 41.2 pg/mL, respectively, p = 0.015). Soluble CD40L and EGF were significantly higher in non-severe cases than in severe cases (sCD40L: 5460.6 pg/mL vs 2643.0 pg/mL, respectively, p<0.001; EGF: 80.1 pg/mL vs 49.5 pg/mL, respectively, p = 0.025) (Fig 4). As for IP-10, some samples presented values above the upper limit of quantification for sCD40L. This factor was then measured again separately, and comparisons are presented in S3 Table. This new evaluation resulted in the same conclusion, i.e. that higher levels of sCD40L were observed in non-severe than in severe dengue cases (median levels 2058.9 pg/mL vs 1144.1 pg/mL, p<0.001). On the other hand, IP-10 new quantification showed higher levels in severe than non-severe cases (median levels 27765.8 pg/mL vs 17493.2 pg/mL, p<0.001) (S2 Table).

### Active versus hospitalization phase

No overall significant differences were found between the CYD-TDV and placebo groups when breakthrough cases in the active and hospitalization phase were analyzed separately (for each cytokine p-value > 0.05) except in the active phase, where sCD40L was significantly higher in CYD-TDV than in placebo recipients (10000 pg/mL [upper range] vs 5311.9 pg/mL, respectively, p = 0.017). In the CYD-TDV group, sCD40L levels dropped significantly in the hospital phase to 2381 pg/mL (p = 0.001), which was also the case in the placebo group dropping to 3203.8 pg/mL although this was non-significant (p = 0.0538). No significant differences were observed for sCD40L between CYD-TDV and placebo in the hospital phase, or between active and hospital phases in the placebo group. The new quantification confirmed above differences with higher levels in vaccinees than in placebos (2437.1 pg/mL vs 1863.6 pg/mL, p = 0.003; S3 Table).

### Participants aged <9 years versus those aged ≥9 years

PLS-DA could not discriminate between the overall immune profile of children aged <9 years and those ≥9 years in the CYD-TDV and placebo groups, with discrimination indices of 23.8% and 12%, respectively (S4A and S4B Fig). Moreover, no discrimination between the two age groups was pointed out (percentage discrimination of 12.7%) regardless of treatment groups. The same was true for the comparison between CYD-TDV and placebo recipients when considering each age group (discrimination indices of 18.9% and 3.8% for <9 years and for ≥9 years respectively).

**Fig 5:**
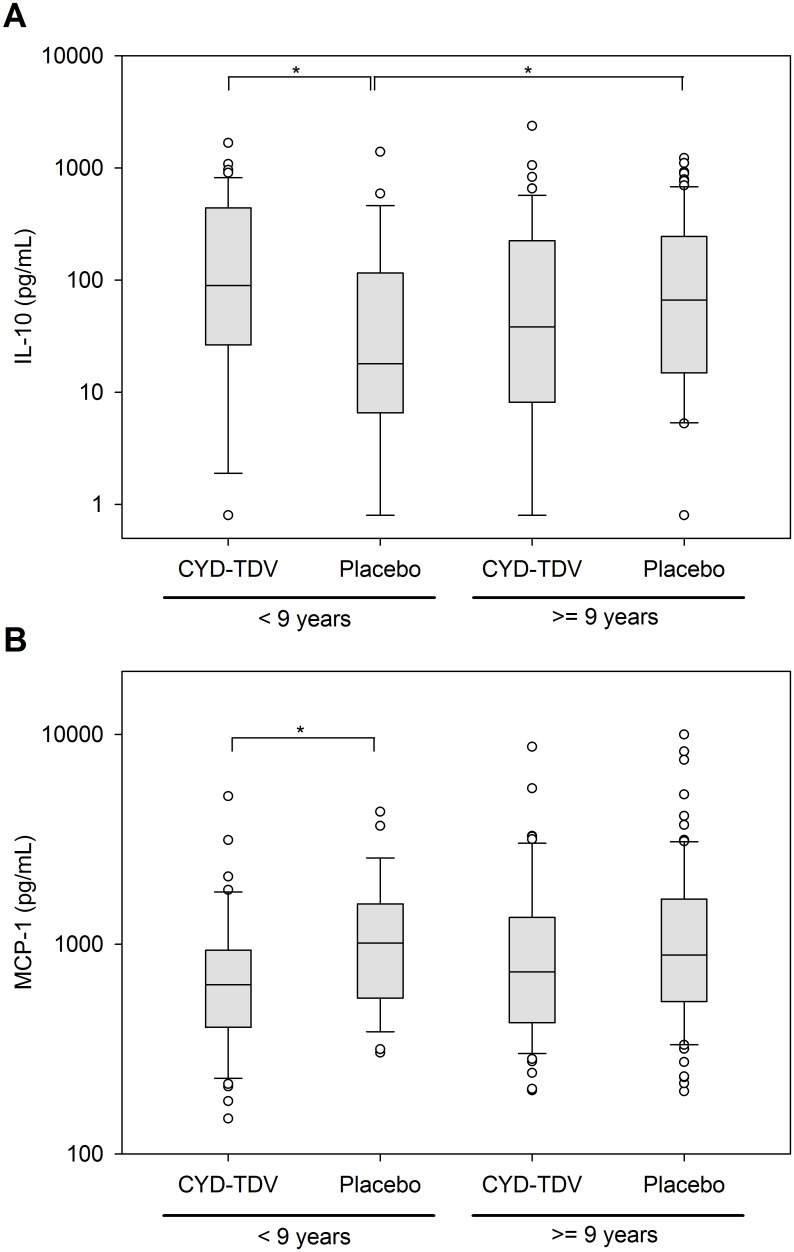
Significant differences in IL-10 and MCP-1 levels between groups among <9 year old children. Box and whisker plots of circulating (A) IL-10 and (B) MCP-1 levels during the acute phase of dengue illness in CYD-TDV and placebo recipients in children <9 years of age (CYD-TDV N = 43, Placebo N = 27) and ≥9 years of age (CYD-TDV N = 56, Placebo N = 81). The boundary of the box closest to zero indicates the 25th percentile, the line within the box marks the median, and the boundary of the box farthest from zero indicates the 75th percentile. Whiskers (error bars) above and below the box indicate the 90th and 10th percentiles. Values below the lower limit of quantification were replaced by half of the limit of quantification. Significant differences are indicated by *p < 0.05.

Each cytokine was also compared individually between the two age groups: Table 1 summarizes those cytokines with significant differences between children aged < 9 years and those aged ≥9 years in the CYD-TDV or placebo groups. In the placebo group, IL-10 levels were significantly lower in children aged <9 years than those aged ≥9 years (p = 0.025). Among children aged <9 years, significant differences in IL-10 (Fig 5A), MCP-1 (Fig 5B) and IFN-α2 levels were observed between treatment groups (Table 1), with higher IL-10 levels (p = 0.037), and lower MCP-1 (p = 0.026) and IFN-α2 (p = 0.006) levels, among CYD-TDV recipients. No significant differences between treatment groups were found among the individual cytokine comparisons for those aged ≥9 years.

**Table 1:** Cytokines with significant differences between children aged < 9 years or ≥ 9 years in the CYD-TDV or placebo groups. Data shown are geometric mean (GM), median, min and max values and the p-values calculated with the Wilcoxon Test (if data were not distributed normally) or Student Test (if data were normally distributed). Significant differences are highlighted in bold.

Cytokines	Group	Age (years)	N	GM (pg/mL)	Median (pg/mL)	Min (pg/mL)	Max (pg/mL)	Wilcoxon Test	Student Test
**Eotaxin**	CYD-TDV	<9	43	85	**94.7**	3.4	459.7	**0.011**	
		≥9	56	115.6	**124.8**	3.4	572.0		
	Placebo	<9	27	116.5	115.3	34.9	318.6		0.859
		≥9	81	119.0	120.4	16.9	772.4		
**EGF**	CYD-TDV	<9	43	47.7	89.0	2.3	417.2	0.633	
		≥9	56	46.8	70.7	2.3	608.1		
	Placebo	<9	27	84.3	**90.8**	17.0	286.9	**0.015**	
		≥9	81	38.9	**60.0**	2.3	473.6		
**IFN-α2**	CYD-TDV	<9	43	5.1	2.4	2.4	127.4	0.907	
		≥9	56	5.0	2.4	2.4	181.4		
	Placebo	<9	27	15.8	**28.5**	2.4	450.5	**0.011**	
		≥9	81	6.0	**2.4**	2.4	371.3		
**IL-10**	CYD-TDV	<9	43	65.4	89.6	0.8	1670.7	0.135	
		≥9	56	33.7	38.4	0.8	2367.9		
	Placebo	<9	27	**21.1**	18.0	0.8	1391.1		**0.025**
		≥9	81	**58.3**	66.5	0.8	1222.7		
**GRO**	CYD-TDV	<9	43	642.0	716.4	105.2	2533.8	0.311	
		≥9	56	354.3	646.8	7.1	2178.1		
	Placebo	<9	27	884.9	**998.4**	85.3	1928.3	**0.010**	
		≥9	81	573.9	**727.6**	7.1	3069.1		
**sCD40L**	CYD-TDV	<9	43	4001.2	5577.8	305.1	10000.0	0.231	
		≥9	56	2736.8	4141.9	64.1	10000.0		
	Placebo	<9	27	5342.5	**10000.0**	230.4	10000.0	**0.008**	
		≥9	81	2922.0	**3983.4**	127.9	10000.0		

### New quantification of IP-10 and sCD40L

As mentioned in above sections, these two factors were above the upper limit of quantification in the first experiment. New tests were then performed to quantify them more accurately, using different kits from the initial test, thus preventing direct comparisons with the former results. Therefore, the different group comparisons were re-assessed as presented in S2 and S3 Tables. As stated previously, these new analyses confirmed and expanded previous conclusions. For each of these factors, two comparisons showed significant differences: i) regarding IP-10, levels were higher in severe cases irrespective of treatment group, and were also higher in placebos *vs* vaccinees when specifically considering severe cases; ii) in contrast, regarding sCD40L, levels were higher in non-severe cases, and were also higher in vaccinees *vs* placebos when specifically considering the active phase.

## Discussion

Thirty-eight cytokines were measured in serum samples from CYD14 and CYD15 study participants during their acute phase of dengue illness. No differences in overall immune profiles were observed between CYD-TDV and placebo recipients regardless of the stratification used for the PLS-DA. This corroborates the absence of any difference in symptomatology, viremia and blood parameters previously reported between the CYD-TDV and placebo groups [16–18]. Of the 15 cytokines with median levels above the LLOQ, only two, IL-1Ra and MCP-1, significantly differed between the treatment groups. Both cytokines were higher in the placebo group. Notably, MCP-1 was also significantly higher in placebo group than in the CYD-TDV group among participants aged <9 years. Moreover, these two cytokines appeared significantly higher in severe cases than non-severe cases in the placebo group.

IL-1Ra is an anti-inflammatory cytokine that can attenuate IL-1-induced fever (by neutralizing IL-1β). It is therefore thought to exert antipyretic action and may be produced as a feedback mechanism to counteract the early increase in IL-1β levels in DF [19]. In a prospective study of children with DSS (n = 50), IL-1Ra and IL-6 plasma concentrations were significantly higher in non-survivors than in survivors [8]. Moreover, IL-1Ra plasma concentrations at day of admission were significantly associated with mortality and were elevated in severe cases.

MCP-1 is a potent chemotactic factor for monocytes and macrophages, both are major sources of MCP-1. This cytokine may increase vascular permeability thereby leading to plasma leakage in dengue patients [20,21]. Higher MCP-1 levels in dengue-infected patients compared to controls have been reported [19,20]. Since IL-1Ra and MCP-1 levels were significantly higher in placebo recipients than CYD-TDV recipients in our study, and both are soluble mediators associated with increased dengue severity, this is a positive finding regarding a potential risk linked to the vaccine.

sCD40L levels were higher during the active phase in the CYD-TDV group, and in non-severe cases irrespective of the treatment group (S3 Table). Soluble CD40L is mainly released by activated platelets and contributes to promote coagulation, therefore higher levels could be beneficial in this regard [22]. A related hypothesis is also put forward by another study proposing that the levels of sCD40L may directly and more significantly reflect thrombocytopenia than platelet counts; in agreement with our findings, the same authors also demonstrating lower levels of sCD40L in severe dengue [23]. sCD40L may then be associated to platelet counts and help counteract hemorrhage in severe dengue cases, in-line with the higher sCD40L levels in non-severe than severe cases in our study. Interestingly, in the case of Ebola hemorrhagic fever, non-fatal cases had higher levels of sCD40L [24], which may be in agreement of the present observation in the case of non-severe dengue. Still in favor of a positive vaccine impact, the opposite finding was seen for IP-10 with levels higher in severe cases and lower in vaccinees than in placebos when specifically considering severe cases. Our findings are also in agreement with another study demonstrating higher levels of IP-10 in severe cases [25].

The lower EGF levels observed in severe cases are also consistent with previous observations of significantly lower mean EGF levels in patients with DHF than DF [26].

Intrinsic ADE was shown in some culture-based studies to suppress IL-12, IFN-γ and TNF-α expression, and to increase the expression of the anti-inflammatory cytokines IL-6 and IL-10 [13,27]. This differential expression of pro- and anti- inflammatory cytokines may play a role in the pathogenesis of severe dengue [28]. In our study, median levels of IL-6 were below the LLOQ in both the CYD-TDV and placebo groups, and the median levels of IL-10 did not differ between the two groups overall (46.8 pg/mL and 58.7 pg/mL, respectively [p>0.05]). In contrast to the overall trend, although IL-10 was significantly higher in children aged <9 years in the CYD-TDV group than the placebo group, this was due to an unexpectedly low level in the placebo group whereas the value observed in the CYD-TDV group was within the range expected. There was no significant difference in IL-10 levels in vaccinated children aged <9 years compared with those ≥9 years or those aged ≥9 years who received placebo. However, the IL-10 levels reported in the current study were lower than those commonly reported in the literature during symptomatic/severe dengue [7,29,30]. The small difference in IL-10 levels in our study is unlikely to explain the imbalance in hospitalization for breakthrough dengue among children aged <9 years between the two groups. In this regard, since IL-10 levels have been shown to be higher in secondary dengue infections than in primary infections or in healthy controls [31], it can be hypothesized that, in children aged <9 years, infections occurring in the CYD-TDV group are akin to secondary infections where vaccination may be considered as the primary infection/challenge and breakthrough disease as secondary infection. Whereas, in the placebo group, the breakthrough disease observed may represent predominantly primary infections [32]. This is supported by the observation that IFN-α2, elevated in primary infections [33], was higher in children aged <9 years in the placebo group.

IL-6 has been shown by several groups to be elevated during dengue illness [7–10] and associated with severe forms of the disease. In our study, only 30% of the participants in the two study groups had levels above the LLOQ, albeit at low to moderate levels. No differences were seen between the two study groups stratified by age, phase of study or severity of disease. In agreement with the similar overall immune profile between CYD-TDV and placebo recipients, no differences were seen in clinical symptoms and viremia levels in hospitalized/severe cases between the two groups [18]. Overall, only two cytokines (IL-1Ra and MCP-1) were significantly higher in the placebo than the CYD-TDV group. Notably, CYD-TDV recipients had higher sCD40L and lower IL-1Ra levels, a profile linked to a milder disease in the present study.

Numerous studies have focused on the identification of cytokines specifically expressed during dengue illness, which could predict the development of severe forms of the disease [34,35]. A limitation of these studies, including our present work, is the fact that samples are often collected at a single time point and at different times of the clinical course of infection (between 1 and 15 days after fever onset). This heterogeneity in sampling time could lead to misleading results due the transient expression of circulating cytokines. Although our study was not designed to control for this variable, sampling times do not appear to be different between the CYD-TDV and placebo groups (sampling occurred at an average of 3.82 and 3.36 days after fever onset, respectively [S1 Table]). Finally, the cytokines that showed significantly elevated levels in the acute samples compared to baseline (IFN-γ, IL-8, TNFα, IL-1α, IL-1Ra, IL-10, FGF-2, eotaxin, MCP-1, MDC, sCD40L, and IP-10) were those expected based on previous findings [7–11,34].

In conclusion, our study confirms that CYD-TDV does not induce an overall altered cytokine profile with breakthrough disease compared with placebo. This is in agreement with the similarities in symptomatology, viremia and blood parameters reported in the two groups.

## Supporting Information

S1 Fig No differences in the overall cytokine profile between groups or trials. Partial least square discriminant analysis (PLS-DA) of acute phase samples (N = 207) on 12 cytokines (eotaxin, FGF-2, IFN-γ, IL-10, IL-1Ra, IL-1α, IL-8, IP-10, MCP-1, MDC, TNF-α and sCD40L). Two-dimension coordinates are plotted: A) by study group (dots corresponding to the placebo group are colored in blue and dots corresponding to the CYD-TDV group are colored in red); and B) by study (dots corresponding to samples from CYD14 study are colored in blue and dots corresponding to samples from CYD15 study are colored in red).
(TIF)

S2 Fig Same kinetics profile between study groups for IL-1RA, MCP-1 and sCD40L. Kinetics profile of the median levels of (A) IL-1Ra and (B) MCP-1 by day after fever onset in CYD-TDV and placebo recipients and in healthy controls (CTRL) (post-dose 3) (N = 20). In the placebo group, 24 participants were sampled at day 1 (D1), 22 at D2, 16 at D3, 17 at D4, 18 at D5, 3 at D6, and 2 at D7. In CYD-TDV group, 20 participants were sampled at day 1 (D1), 14 at D2, 15 at D3, 19 at D4, 13 at D5, 4 at D6, and 7 at D7. Upper error bars represent the 75% (Q3) percentile; the lower error bars represent the 25% (Q1) percentile. Values below the lower limit of quantification were replaced by half of the limit of quantification. The dotted line represents the lower limit of quantification (LLOQ). *ND = not determined since not enough sample volume was available to allow a retest.
(TIF)

S3 Fig IFN-α2 and IL-15 significantly lower in vaccinees in severe cases. Box and whisker plots of circulating IFN-α2 (left panel) and IL-15 (right panel) levels during the acute phase of dengue illness in CYD-TDV and placebo recipients: (A) irrespective of severity (CYD-TDV N = 99, Placebo N = 108); (B) in severe cases (CYD-TDV N = 24, Placebo N = 28); and (C) in non-severe cases (CYD-TDV N = 74, Placebo N = 80). The boundary of the box closest to zero indicates the 25th percentile, the line within the box marks the median, and the boundary of the box farthest from zero indicates the 75th percentile. Whiskers (error bars) above and below the box indicate the 90th and 10th percentiles. The individual points represent outliers. Values below the lower limit of quantification were replaced by half of the limit of quantification. Significant differences are indicated by *p < 0.05, **p ≤ 0.01 and ***p ≤ 0.001.
(TIF)

S4 Fig No difference in overall immune profile of children aged <9 years and those aged ≥ 9 years. Partial least square discriminant analysis (PLS-DA) of acute phase samples on 12 cytokines (eotaxin, FGF-2, IFN-γ, IL-10, IL-1Ra, IL-1α, IL-8, IP-10, MCP-1, MDC, TNF-α and sCD40L) for the CYD-TDV and placebo groups. Two-dimension coordinates are plotted: A) CYD-TDV group, dots corresponding to children aged <9 years are colored in blue (N = 43) and dots corresponding to children ≥9 years are colored in red (N = 56); B) placebo group, dots corresponding to children aged <9 years are colored in blue (N = 27) and dots corresponding to children ≥9 years are colored in red (N = 81).
(TIF)

S1 Table Demographic characteristics of hospitalized participants with virologically confirmed dengue across both clinical trials (CYD14 and CYD15). (DOCX)

S2 Table New quantification and comparisons for IP-10. (DOCX)

S3 Table New quantification and comparisons for sCD40L. (DOCX)
