# Supplementary figures and images for: Cytokine Profile of Children Hospitalized with Virologically-Confirmed Dengue during Two Phase III Vaccine Efficacy Trials
Source: PLoS Negl Trop Dis. 2016 Jul 26;10(7):e0004830. doi: 10.1371/journal.pntd.0004830 (PMC4961416; doi:10.1371/journal.pntd.0004830)

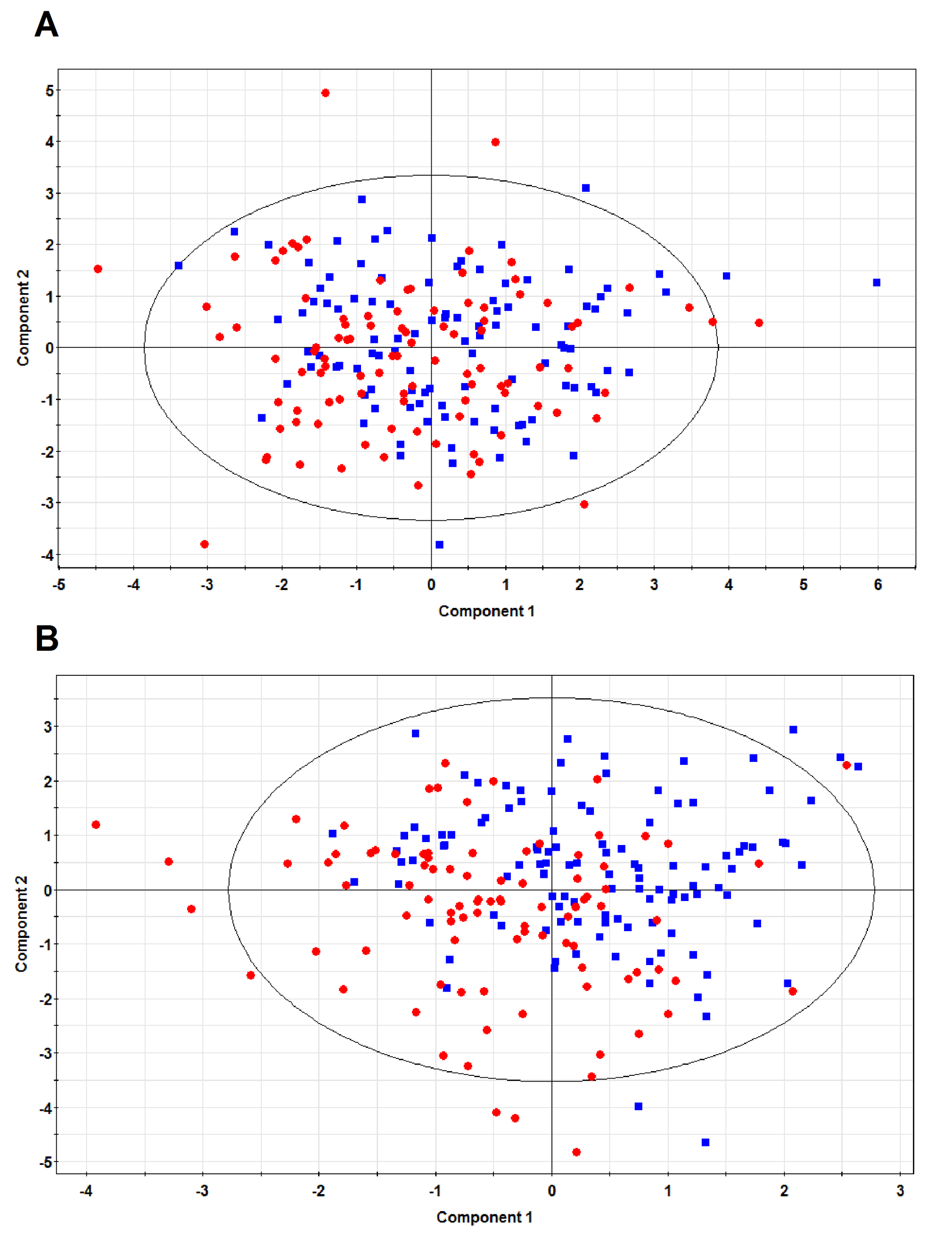

Supplement: S1 Fig — Partial least square discriminant analysis (PLS-DA) of acute phase samples (N = 207) on 12 cytokines (eotaxin, FGF-2, IFN-γ, IL-10, IL-1Ra, IL-1α, IL-8, IP-10, MCP-1, MDC, TNF-α and sCD40L). Two-dimension coordinates are plotted: A) by study group (dots corresponding to the placebo group are colored in blue and dots corresponding to the CYD-TDV group are colored in red); and B) by study (dots corresponding to samples from CYD14 study are colored in blue and dots corresponding to samples from CYD15 study are colored in red). (TIF) [file pntd.0004830.s001.tif]

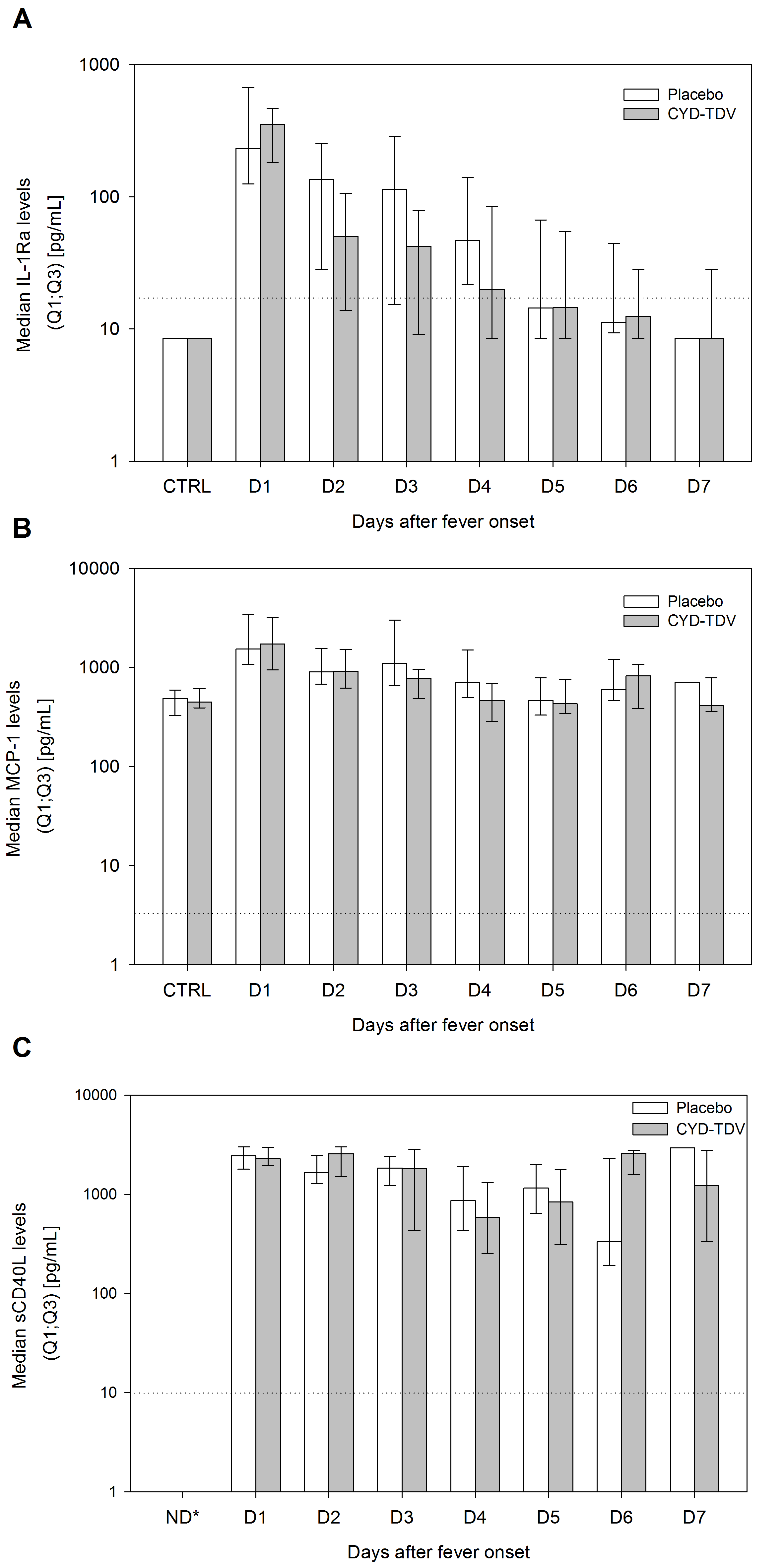

Supplement: S2 Fig — Kinetics profile of the median levels of (A) IL-1Ra and (B) MCP-1 by day after fever onset in CYD-TDV and placebo recipients and in healthy controls (CTRL) (post-dose 3) (N = 20). In the placebo group, 24 participants were sampled at day 1 (D1), 22 at D2, 16 at D3, 17 at D4, 18 at D5, 3 at D6, and 2 at D7. In CYD-TDV group, 20 participants were sampled at day 1 (D1), 14 at D2, 15 at D3, 19 at D4, 13 at D5, 4 at D6, and 7 at D7. Upper error bars represent the 75% (Q3) percentile; the lower error bars represent the 25% (Q1) percentile. Values below the lower limit of quantification were replaced by half of the limit of quantification. The dotted line represents the lower limit of quantification (LLOQ). *ND = not determined since not enough sample volume was available to allow a retest. (TIF) [file pntd.0004830.s002.tif]

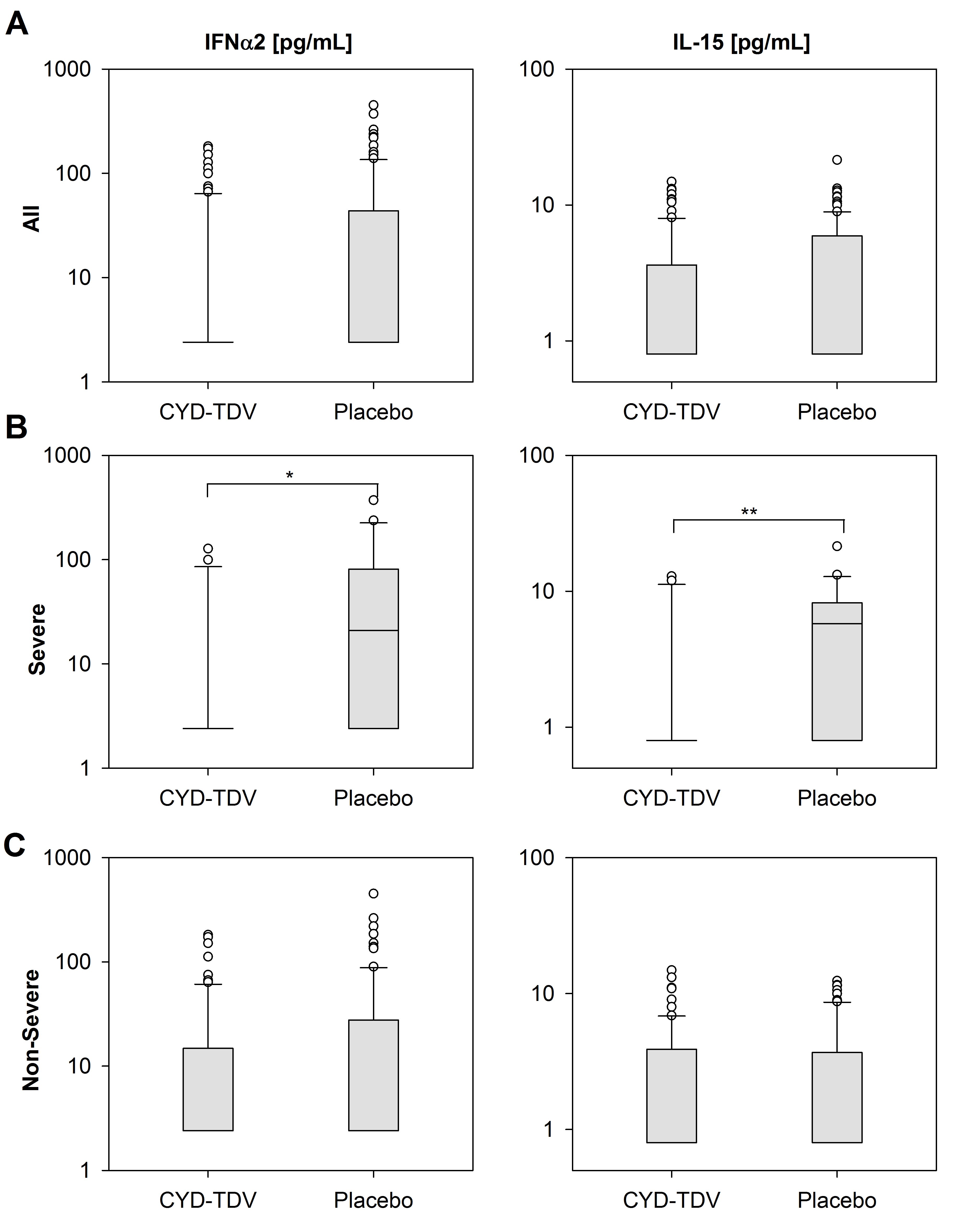

Supplement: S3 Fig — Box and whisker plots of circulating IFN-α2 (left panel) and IL-15 (right panel) levels during the acute phase of dengue illness in CYD-TDV and placebo recipients: (A) irrespective of severity (CYD-TDV N = 99, Placebo N = 108); (B) in severe cases (CYD-TDV N = 24, Placebo N = 28); and (C) in non-severe cases (CYD-TDV N = 74, Placebo N = 80). The boundary of the box closest to zero indicates the 25th percentile, the line within the box marks the median, and the boundary of the box farthest from zero indicates the 75th percentile. Whiskers (error bars) above and below the box indicate the 90th and 10th percentiles. The individual points represent outliers. Values below the lower limit of quantification were replaced by half of the limit of quantification. Significant differences are indicated by *p < 0.05, **p ≤ 0.01 and ***p ≤ 0.001. (TIF) [file pntd.0004830.s003.tif]

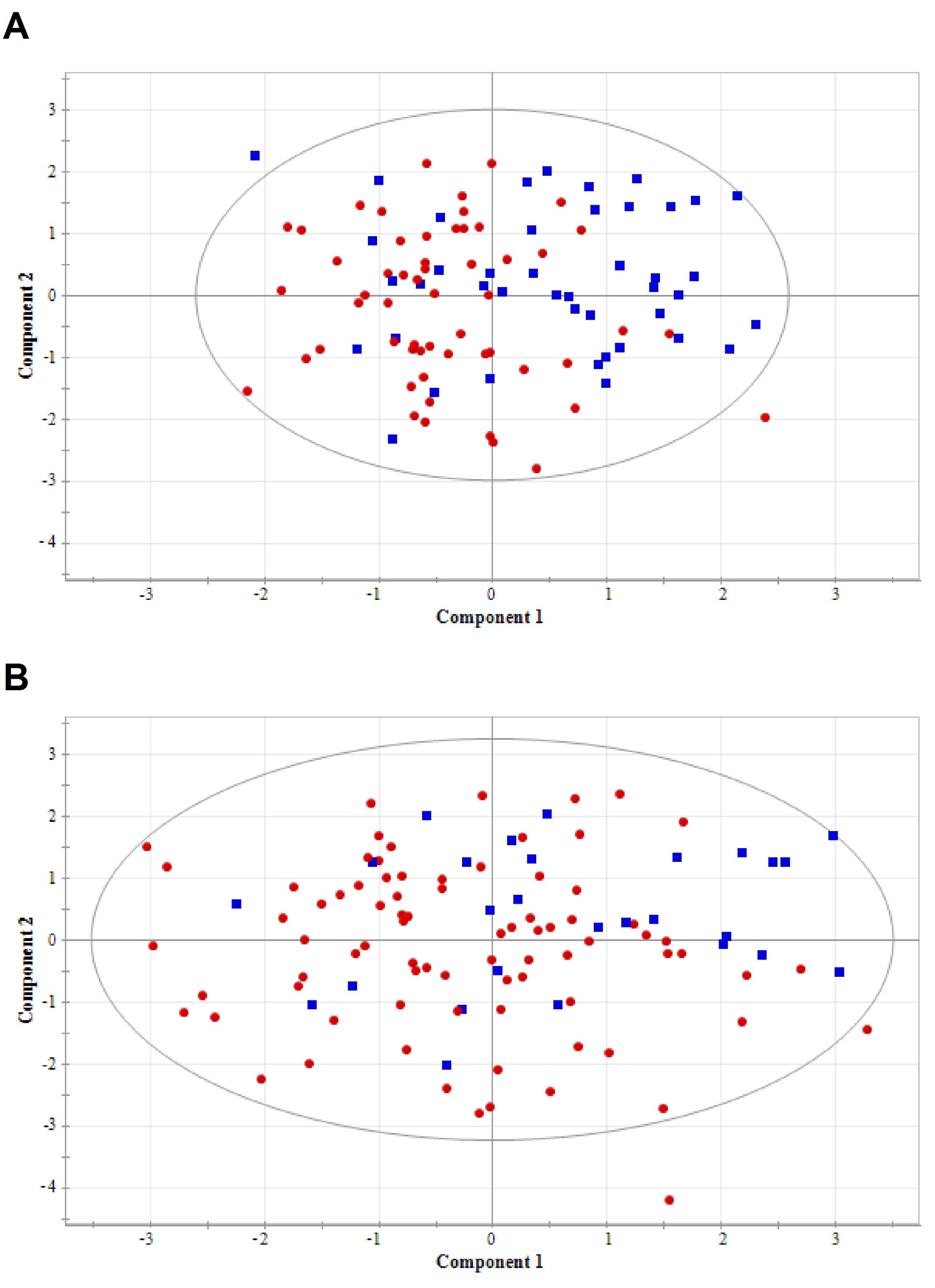

Supplement: S4 Fig — Partial least square discriminant analysis (PLS-DA) of acute phase samples on 12 cytokines (eotaxin, FGF-2, IFN-γ, IL-10, IL-1Ra, IL-1α, IL-8, IP-10, MCP-1, MDC, TNF-α and sCD40L) for the CYD-TDV and placebo groups. Two-dimension coordinates are plotted: A) CYD-TDV group, dots corresponding to children aged <9 years are colored in blue (N = 43) and dots corresponding to children ≥9 years are colored in red (N = 56); B) placebo group, dots corresponding to children aged <9 years are colored in blue (N = 27) and dots corresponding to children ≥9 years are colored in red (N = 81). (TIF) [file pntd.0004830.s004.tif]
