# Supplementary material for: Cytokine Profile of Children Hospitalized with Virologically-Confirmed Dengue during Two Phase III Vaccine Efficacy Trials
Source: PLoS Negl Trop Dis. 2016 Jul 26;10(7):e0004830. doi: 10.1371/journal.pntd.0004830 (PMC4961416; doi:10.1371/journal.pntd.0004830)
Supplement: S1 Table — (DOCX) [file pntd.0004830.s005.docx]

**Supplementary Table 1:** Demographic characteristics of hospitalized participants with virologically confirmed dengue across both clinical trials (CYD14 and CYD15).

|  | | **CYD Dengue Vaccine Group** | **Control Group** | **Total** |
| --- | --- | --- | --- | --- |
| **All particpants** | **Number of subjects** | 99 | 108 | 207 |
|  | **Age Mean (Min; Max)** | 8.82 (2.15; 14.9) | 10.6 (2.22; 16.9) | 9.76 (2.15; 16.9) |
|  | **< 9 years** | 43 | 27 | 70 |
|  | **>= 9 years** | 56 | 81 | 137 |
|  | **3 injections** | 98 | 107 | 205 |
|  | **Acute sample date - Start date of the case: Mean (Min; Max)** | 3.82 (1.00; 14.0) | 3.36 (1.00; 15.0) | 3.58 (1.00; 15.0) |
| **Active phase** | **Number of subjects** | 42 | 76 | 118 |
|  | **Age Mean (Min; Max)** | 9.17 (2.15; 14.7) | 10.3 (2.22; 16.3) | 9.90 (2.15; 16.3) |
|  | **< 9 years** | 17 | 22 | 39 |
|  | **>= 9 years** | 25 | 54 | 79 |
|  | **3 injections** | 41 | 75 | 116 |
|  | **Acute sample date - Start date of the case: Mean (Min; Max)** | 2.74 (1.00; 14.0) | 2.60 (1.00; 8.00) | 2.65 (1.00; 14.0) |
| **Hospital phase** | **Number of subjects** | 56 | 32 | 88 |
|  | **Age Mean (Min; Max)** | 8.49 (2.35; 14.9) | 11.4 (5.19; 16.9) | 9.55 (2.35; 16.9) |
|  | **< 9 years** | 26 | 5 | 31 |
|  | **>= 9 years** | 30 | 27 | 57 |
|  | **3 injections** | 56 | 32 | 88 |
|  | **Acute sample date - Start date of the case: Mean (Min; Max)** | 4.63 (1.00; 12.0) | 5.16 (2.00; 15.0) | 4.82 (1.00; 15.0) |
| **Severe (IDMC assessment)** | **Number of subjects** | 24 | 28 | 52 |
|  | **Age Mean (Min; Max)** | 7.30 (2.15; 14.0) | 10.9 (4.21; 16.9) | 9.26 (2.15; 16.9) |
|  | **< 9 years** | 14 | 6 | 20 |
|  | **>= 9 years** | 10 | 22 | 32 |
|  | **3 injections** | 24 | 28 | 52 |
|  | **Acute sample date - Start date of the case: Mean (Min; Max)** | 4.42 (1.00; 12.0) | 3.21 (1.00; 14.0) | 3.77 (1.00; 14.0) |
| **Non-severe (IDMC assessment): NO and OTHER** | **Number of subjects** | 74 | 80 | 154 |
|  | **Age Mean (Min; Max)** | 9.26 (2.35; 14.9) | 10.5 (2.22; 16.4) | 9.92 (2.22; 16.4) |
|  | **< 9 years** | 29 | 21 | 50 |
|  | **>= 9 years** | 45 | 59 | 104 |
|  | **3 injections** | 73 | 79 | 152 |
|  | **Acute sample date - Start date of the case: Mean (Min; Max)** | 3.62 (1.00; 14.0) | 3.42 (1.00; 15.0) | 3.52 (1.00; 15.0) |

IDMC, independent data monitoring committee
