# Supplementary material for: Cytokine Profile of Children Hospitalized with Virologically-Confirmed Dengue during Two Phase III Vaccine Efficacy Trials
Source: PLoS Negl Trop Dis. 2016 Jul 26;10(7):e0004830. doi: 10.1371/journal.pntd.0004830 (PMC4961416; doi:10.1371/journal.pntd.0004830)
Supplement: S2 Table — (DOCX) [file pntd.0004830.s006.docx]

**Supplementary Table 2:** New quantification and comparisons for IP-10

| **IP-10** | **Group** | **N** | **GM**  **(pg/mL)** | **Median**  **(pg/mL)** | **Min**  **(pg/mL)** | **Max**  **(pg/mL)** | **Wilcoxon Test** |
| --- | --- | --- | --- | --- | --- | --- | --- |
| CYD-TDV versus placebo for all hospitalized cases | CYD-TDV | 95 | 15433.4 | 19633.8 | 7.0 | 121009.6 | 0.177 |
|  | Placebo | 104 | 19413.1 | 19273.6 | 994.6 | 200618.2 |  |
| CYD-TDV versus placebo for severe cases | CYD-TDV | 24 | 18707.9 | **21648.2** | 3511.2 | 56918.3 | **0.033** |
|  | Placebo | 26 | 31246.1 | **40288.0** | 2120.4 | 87711.0 |  |
| CYD-TDV versus placebo for non-severe cases | CYD-TDV | 70 | 14356.2 | 18104.7 | 7.0 | 121009.6 | 0.763 |
|  | Placebo | 78 | 16565.1 | 17232.4 | 994.6 | 200618.2 |  |
| Severe versus non-severe cases irrespective of treatment group | Severe | 50 | 24426.7 | **27765.8** | 2120.4 | 87711.0 | **<0.001** |
|  | Non-severe | 148 | 15480.9 | **17493.2** | 7.0 | 200618.2 |  |
| CYD-TDV versus placebo for active phase | CYD-TDV | 40 | 13549.7 | 21536.3 | 7.0 | 87584.2 | 0.097 |
|  | Placebo | 72 | 22280.1 | 25910.2 | 999.4 | 200618.2 |  |
| CYD-TDV versus placebo for hospital phase | CYD-TDV | 54 | 16855.6 | 16575.6 | 3147.9 | 121009.6 | 0.388 |
|  | Placebo | 32 | 14239.5 | 14109.5 | 1000.8 | 93282.3 |  |
| <9 years versus >=9 years for CYD-TDV group | <9 | 42 | 15209.8 | 18050.0 | 7.0 | 56918.3 | 0.762 |
|  | >=9 | 53 | 15612.9 | 20919.4 | 1087.4 | 121009.6 |  |
| <9 years versus >=9 years for placebo group | <9 | 27 | 20259.8 | 19539.8 | 994.6 | 85569.6 | 0.824 |
|  | >=9 | 77 | 19124.7 | 19178.7 | 1000.8 | 200618.2 |  |
