# Supplementary material for: Cytokine Profile of Children Hospitalized with Virologically-Confirmed Dengue during Two Phase III Vaccine Efficacy Trials
Source: PLoS Negl Trop Dis. 2016 Jul 26;10(7):e0004830. doi: 10.1371/journal.pntd.0004830 (PMC4961416; doi:10.1371/journal.pntd.0004830)
Supplement: S3 Table — (DOCX) [file pntd.0004830.s007.docx]

**Supplementary Table 3:** New quantification and comparisons for sCD40L

| **sCD40L** | **Group** | **N** | **GM**  **(pg/mL)** | **Median**  **(pg/mL)** | **Min**  **(pg/mL)** | **Max**  **(pg/mL)** | **Wilcoxon Test** |
| --- | --- | --- | --- | --- | --- | --- | --- |
| CYD-TDV versus placebo for all hospitalized cases | CYD-TDV | 95 | 1221.9 | 1879.3 | 5.0 | 8263.6 | 0.849 |
|  | Placebo | 104 | 1344.9 | 1733.7 | 113.3 | 5378.3 |  |
| CYD-TDV versus placebo for severe cases | CYD-TDV | 24 | 614.8 | 703.4 | 5.0 | 4939.8 | 0.264 |
|  | Placebo | 26 | 978.0 | 1470.2 | 113.3 | 5378.3 |  |
| CYD-TDV versus placebo for non-severe cases | CYD-TDV | 70 | 1582.2 | 2130.4 | 95.6 | 8263.6 | 0.287 |
|  | Placebo | 78 | 1495.5 | 1822.5 | 157.7 | 4612.6 |  |
| Severe versus non-severe cases irrespective of treatment group | Severe | 50 | 782.7 | **1144.1** | 5.0 | 5378.3 | **<0.001** |
|  | Non-severe | 148 | 1535.9 | **2058.9** | 95.6 | 8263.6 |  |
| CYD-TDV versus placebo for active phase | CYD-TDV | 40 | 2111.6 | **2437.1** | 132.5 | 8263.6 | **0.003** |
|  | Placebo | 72 | 1502.0 | **1863.6** | 157.7 | 5378.3 |  |
| CYD-TDV versus placebo for hospital phase | CYD-TDV | 54 | 839.4 | 1023.1 | 5.0 | 6404.3 | 0.420 |
|  | Placebo | 32 | 1048.9 | 1424.1 | 113.3 | 4612.6 |  |
| <9 years versus >=9 years for CYD-TDV group | <9 | 42 | 1315.3 | 1825.8 | 188.5 | 5069.3 | 0.985 |
|  | >=9 | 53 | 1152.5 | 1901.1 | 5.0 | 8263.6 |  |
| <9 years versus >=9 years for placebo group | <9 | 27 | 1807.2 | 2103.0 | 250.3 | 4612.6 | 0.051 |
|  | >=9 | 77 | 1212.5 | 1602.4 | 113.3 | 5378.3 |  |
